# In-Hospital Mortality from Spondylodiscitis: Insights from a Single-Center Retrospective Study

**DOI:** 10.3390/jcm12237228

**Published:** 2023-11-22

**Authors:** Ann-Kathrin Joerger, Carolin Albrecht, Nicole Lange, Bernhard Meyer, Maria Wostrack

**Affiliations:** Department of Neurosurgery, Klinikum Rechts der Isar, Technical University Munich, 81675 Munich, Germanymaria.wostrack@tum.de (M.W.)

**Keywords:** in-hospital death spondylodiscitis, vertebral osteomyelitis, spondylodiscitis, spinal infection

## Abstract

(1) Background: There is a marked proportion of spondylodiscitis patients who die during the early stage of the disease despite the applied therapy. This study investigates this early mortality and explores the associated risk factors. (2) Methods: We conducted a retrospective analysis of spondylodiscitis patients treated at our Level I spine center between 1 January 2018 and 31 December 2022. (3) Results: Among 430 patients, 32 (7.4%) died during their hospital stay, with a median time of 28.5 days (range: 2.0–84.0 days). Six of these patients (18.75%) did not undergo surgery due to dire clinical conditions or death prior to scheduled surgery. Identified causes of in-hospital death included multiorgan failure (*n* = 15), acute bone marrow failure (2), cardiac failure (4), liver failure (2), acute respiratory failure (2), acute renal failure (1), and concomitant oncological disease (1). In a simple logistic regression analysis, advanced age (*p* = 0.0006), diabetes mellitus (*p* = 0.0002), previous steroid medication (*p* = 0.0279), Charlson Comorbidity Index (*p* < 0.0001), and GFR level at admission (*p* = 0.0008) were significant risk factors for in-hospital death. In a multiple logistic regression analysis, advanced age (*p* = 0.0038), diabetes mellitus (*p* = 0.0002), and previous steroid medication (*p* = 0.0281) remained significant. (4) Conclusions: Despite immediate treatment, a subset of spondylodiscitis patients experience early mortality. Particular attention should be given to elderly patients and those with diabetes or a history of steroid medication, as they face an elevated risk of a rapidly progressing and fatal disease.

## 1. Introduction

Spondylodiscitis—the infection of the intervertebral disc and the adjacent vertebral bodies—is an infectious disease associated with high morbidity and mortality [1]. It primarily affects patients aged 70 years or older [1]. Men are 1.5 times more likely to be affected than women [1]. The incidence of spondylodiscitis is on the rise in Europe as well as in the United States, leading to a high socioeconomic burden [1,2,3]. Notably, in Germany, the incidence rose from 10.4 per 100,000 inhabitants in 2010 to 14.4 per 100,000 in 2020 [1]. In the United States, the incidence increased from 2.9 per 100,000 in 1998 to 5.4 per 100,000 in 2013 [2]. This trend can be attributed to the shifting demographic landscape, encompassing an aging population [4], and to an increase in multimorbid and immunocompromised patients [5,6]. Additionally, better diagnostic techniques and broader access to these diagnostic modalities have also contributed to the enhanced detection of spondylodiscitis cases [7]. Spondylodiscitis may arise through different mechanisms, including hematogenous spread from a distant focus of infection, dissemination from nearby tissue, or direct inoculation [8]. When spondylodiscitis is caused by hematogenous spread, it predominantly affects the lumbar spine (58%), followed by the thoracic (30%) and cervical spine (11%) [9]. In cases of hematogenous spread, the infection can extend beyond the bony structures and affect the surrounding tissue. In contrast, spondylodiscitis originating from dissemination from contiguous tissues is rare and is typically associated with adjacent infections such as esophageal fistula [10]. The mode of direct external inoculation is primarily iatrogenic, often arising from infiltrations or prior surgeries [11]. Although diagnostic and therapeutic guidelines have been established to optimize the management of patients with spondylodiscitis [12,13], delay of diagnosis due to the lack of specific symptoms is still a relevant problem [8]. Additionally, therapy for spondylodiscitis is not standardized and differs locally. The duration and method of antibiotic treatment, as well as the effectiveness of conservative versus surgical treatment, are still matters of debate. While numerous studies report favorable outcomes with low relapse and therapy failure rates [14], there is still a marked proportion of patients who die during the early stage of the disease despite adequate therapy [15]. Older age, diabetes, hemodialysis, endocarditis, malignant diseases, and liver cirrhosis have been identified as risk factors for the death of patients with spondylodiscitis [16]. However, the literature data on these severe cases are sparse. Understanding the present epidemiology and clinical characteristics of these severe cases is of utmost importance to ensure effective treatment. This study’s aim was to describe the clinical data of patients with spondylodiscitis-related early mortality and to identify the associated risk factors.

## 2. Materials and Methods

### 2.1. Population

We conducted a retrospective study comprising consecutive patients treated for spondylodiscitis of the cervical, thoracic, or lumbosacral spine at a high-volume Level I surgical spine center between 1 January 2018 and 31 December 2022. Patients aged 18 years or older were included. Spondylodiscitis cases were retrieved from the hospital’s database by screening for the International Classification of Diseases, Tenth Revision (ICD-10) code for spondylodiscitis. Patients’ records, imaging data, laboratory and microbiological results, and surgical reports were analyzed. The following variables were extracted: age, sex, body mass index (BMI), American Association of Anesthesiologists (ASA) risk classification score, substance abuse, previous steroid medication, comorbidities, (previous or current) malignant disease, hepatopathy, diabetes mellitus, Charlson Comorbidity Index (CCI), C-reactive protein (CRP) level, neurologic status at admission and discharge, infective focus, bacteria cultivated, surgical strategy, and complications. The primary outcome measured was in-hospital mortality. Patients were divided into those with early mortality, i.e., who died during the same hospital stay, and those who survived the hospital stay. Patients who died after discharge from the hospital were excluded.

### 2.2. Diagnostics and Treatment

The diagnosis of spondylodiscitis was established based on the typical clinical presentation of the patients, along with the characteristic radiological features, and confirmed by positive pathogen detection in blood cultures or through CT-guided tissue biopsy. Culture-negative, mycobacterial, or fungal cases were also included in our analysis. The date of the hospitalization was assigned as the date of the diagnosis.

Magnetic resonance imaging (MRI) scans of the whole spine were performed in all patients. Computer tomography (CT) and/or positron emission tomography (PET) were performed if the MRI results were not conclusive enough or in patients who were not able to undergo MRI. 

Septic condition at admission was defined by fulfilling at least two Systemic Inflammatory Response Syndrome (SIRS) criteria: body temperature over 38 or under 36 degrees Celsius; heart rate greater than 90 beats/minute; respiratory rate greater than 20 breaths/minute or partial pressure of CO_2_ less than 32 mmHg; and leucocyte count >12,000/mm^3^ or <4000/mm^3^, or >10% bands.

In almost all cases, surgical therapy was performed except for severely affected patients with very high perioperative risks or patients who died prior to surgery. Surgery typically involved dorsal fixation with pedicle screws and decompression of the infected intervertebral disc space from the dorsal or ventral/lateral approach, followed by fusion using a cage, and local antibiotic application. Empirical antibiotic therapy, usually consisting of a combination of vancomycin and meropenem, was initiated promptly after biopsy collection or, in cases of severely affected patients with elevated inflammation markers, after the collection of blood cultures. Subsequently, antibiotic therapy was tailored to the specific spectrum of identified pathogens. All patients received a minimum of two weeks of intravenous antibiotic therapy, followed by a long-term oral regimen to complete a total of 12 weeks of antibiotic treatment. 

### 2.3. Statistics

Simple logistic regression and multiple logistic regression analysis were used to identify risk factors for in-hospital mortality. A paired t-test (for continuous variables), Fisher’s exact test (for categorical variables), and chi-square test (for categorical variables) were used to compare the clinical data of patients who died during the same hospital stay and of those who survived. Statistical analysis was performed with GraphPad Prism 10.0.2 (Boston, MA, USA). 

## 3. Results

### 3.1. Demographic Background

Over the course of a five-year study period, we enrolled 430 patients with a median age of 72 years (ranging from 30 to 94 years) in our research cohort. Among these participants, 271 (63.0%) were male and 159 (37.0%) were female (Table 1). In total, 32 (7.4%) patients died during the same hospital stay (early mortality group) after a median time of 28.5 days (2.0–84.0 days), while 14 (3.3%) died after being discharged from hospital after a median time of 313 days (54–990 days). These 14 patients were subsequently excluded from further analysis. Consequently, 384 patients (98.3%) successfully survived throughout the study period, with a median follow-up duration of 86 days (ranging from 20 to 1232 days). The median hospital stay for surviving patients was 14 days (2–110 days). This was primarily determined by the duration of intravenous antibiotics.

Notably, the patients in the early mortality group were significantly older than those who survived (*p* = 0.0018) (Table 1). Furthermore, there were significant disparities in the American Association of Anesthesiologists (ASA) risk classification scores between the two groups (*p* < 0.0001). Specifically, patients in the early mortality group were more likely to have a more severe ASA score (IV and V) compared to their counterparts who survived. Additionally, the early mortality group exhibited a notably higher Charlson Comorbidity Index (CCI) compared to those who survived (*p* < 0.0001).

While both groups were comparable regarding body mass index (BMI), alcohol abuse, IV drug abuse, hepatopathy, and history of malignant diseases, the proportion of patients on steroid medication (*p* = 0.0242) and with diabetes mellitus (*p* = 0.0004) was significantly higher in the early mortality group (Table 1). 

### 3.2. Characterization of Spondylodiscitis

**Figure 1:**
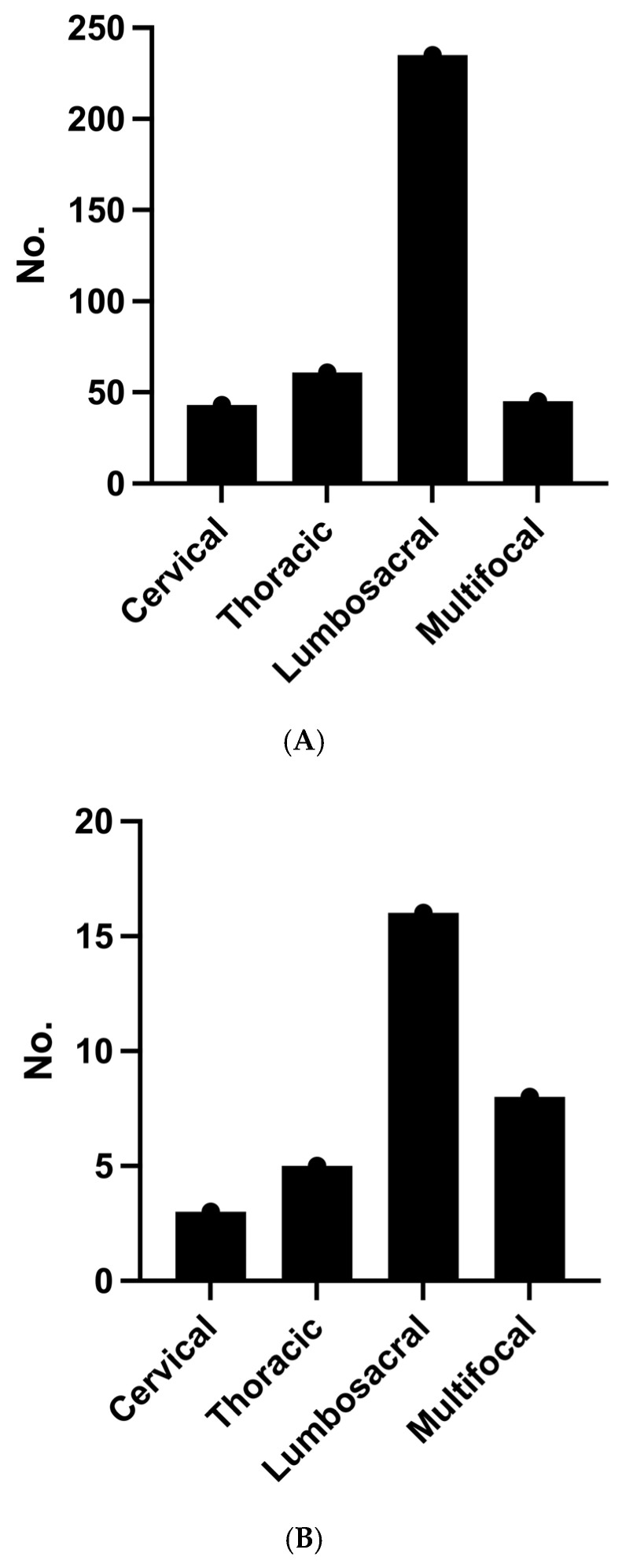
(**A**): Localization of spondylodiscitis in the survivor group. Cervical: *n* = 43; thoracic: *n* = 61; lumbosacral: *n* = 235; multifocal: *n* = 45. (**B**): Localization of spondylodiscitis in early mortality group. Cervical: *n* = 3; thoracic: *n* = 5; lumbosacral: *n* = 16; multifocal: *n* = 8.

In most instances, spondylodiscitis was acquired endogenously, indicating that it occurred without any prior spinal surgery on the same spinal level (Table 2). For both groups, the lumbosacral region emerged as the predominant site for spondylodiscitis (Figure 1A,B). However, it is noteworthy that 25.0% of patients who experienced early mortality had multifocal spondylodiscitis, whereas this occurred in only 11.7% of patients who survived (*p* = 0.0477). Among the surviving patients, there was a trend, albeit statistically non-significant, towards less frequent occurrences of epidural empyema and paravertebral abscess compared to patients with early mortality (Table 2). 

C-reactive protein (CRP) level at admission was significantly higher for patients who died during the same hospital stay. 

Interestingly, only five (15.6%) patients experiencing early mortality were admitted in a clinically compromised state necessitating intensive care therapy. The vast majority (84.4%) initially presented in a stable general condition but experienced rapid deterioration during their hospitalization. Of the surviving patients, 20.8% had sepsis at admission and 10.2% suffered from sepsis-induced hepatopathy. Of patients with in-hospital mortality, 50.0% had sepsis when admitted to hospital and 12.5% suffered from hepatopathy. While almost all surviving patients were treated surgically, 18.8% of patients in the early mortality group did not receive surgical treatment due to their swift deterioration and death before scheduled surgery.

Table 3 provides an overview of patients’ neurological status at admission. Patients with early mortality were more likely to exhibit significant motor deficits (as indicated by a Medical Research Council Scale for Muscle Strength score of 3 or less) (*p* = 0.0064) or bowel/bladder dysfunction (*p* = 0.0054) compared to those who survived.

### 3.3. Focus of Spondylodiscitis and Microbiology

Among patients in the early mortality group, the most prevalent sources of infection were joint empyema (21.9%), urosepsis (18.8%), and leg ulcers (15.6%), respectively (Table 4). In contrast, for surviving patients, the primary source of infection was prior spinal surgery (29.4%) (Table 5). Notably, 4 out of 32 patients (12.5%) in the early mortality group were afflicted with endocarditis (Table 4), in comparison to 19 out of 384 surviving patients (4.9%), although this difference was not statistically significant. Patients with endocarditis in the early mortality group exhibited a significantly worse ASA score than those without endocarditis in the same group (*p* = 0.0138).

**Figure 2:**
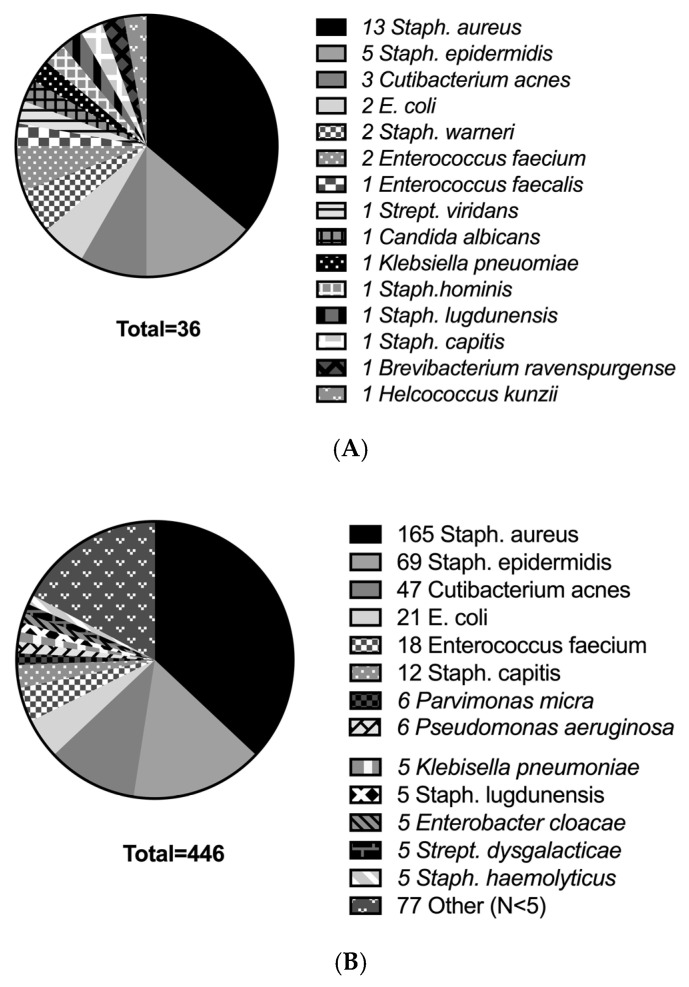
(**A**). Microbiological results of the early mortality group. (**A**) shows the results of microbiological cultivation (blood cultures and tissue) in the early mortality group. E. = *Eschericha*; *Staph.* = *Staphylococcus*; *Strept.* = *Streptococcus.* (**B**). Microbiological results of the survivor group. (**B**) shows the results of microbiological cultivation (blood cultures and tissue) in the survivor group. For the sake of clarity, bacteria that were detected fewer than 5 times were grouped under the category “Other”. E. = *Eschericha*; *Staph.* = *Staphylococcus*; *Strept.* = *Streptococcus*.

Bacterial cultures of infected disc tissue were successfully obtained in 78.9% of surviving patients and 81.3% of patients with early mortality, respectively. In both groups, the most frequently isolated pathogen was *Staphylococcus aureus*, followed by *Staphylococcus epidermidis* and *Cutibacterium acnes* (Figure 2A,B). *Mycobacterium tuberculosis* was found in three cases (0.8%) among surviving patients and in none of the patients with early mortality. A fungal infection with *Candida albicans* was detected in three cases (0.8%) among the surviving patients, whereas in patients with early mortality, it was observed in one case (3.1%).

### 3.4. In-Hospital Mortality: Causes and Risk Factors

The overall in-hospital mortality rate was 7.4%, comprising 32 out of 430 patients. The documented causes of in-hospital mortality were multiorgan failure (*n* = 15), acute bone marrow failure (*n* = 2), cardiac failure (*n* = 4), cirrhosis-related liver failure (*n* = 2), acute respiratory failure (*n* = 2), acute renal failure (*n* = 1), and the presence of concomitant oncological disease (*n* = 1). In five cases, a single specific cause of death could not be identified.

In a simple logistic regression analysis, advanced age (*p* = 0.0006), diabetes mellitus (*p* = 0.0002), previous steroid medication (*p* = 0.0279), Charlson Comorbidity Index (CCI) (*p* < 0.0001), and glomerular filtration rate (GFR) level at admission (*p* = 0.0008) were significant risk factors for in-hospital death (Supplementary Table S1). However, variables such as BMI, CRP level at admission, history of malignant disease, alcohol abuse, hepatopathy, endocarditis, paraspinal abscess, and intraspinal empyema did not reach statistical significance. A multiple logistic regression analysis identified advanced age (*p* = 0.0038), diabetes mellitus (*p* = 0.0002), and previous steroid medication (*p* = 0.0281) as significant risk factors for in-hospital death (Supplementary Table S2).

## 4. Discussion

This study aimed to investigate the rate of in-hospital mortality in patients with spondylodiscitis and explore its associated risk factors. 

Over the five-year study period, the in-hospital mortality rate was 7.4% (32 out of 430). This rate is consistent with findings from a large-scale Japanese study (6%) [16] and a smaller study conducted in the United Kingdom (7%) [17]. However, recent German studies reported even higher in-hospital mortality rates ranging from 10 to 14% [15,18,19,20]. This persistently high mortality underscores that spondylodiscitis remains a potentially life-threatening disease, despite advancements in diagnostic and treatment approaches. It should be recognized and treated not merely as a localized infection of bones and intervertebral discs but also as a systemic disease. As a result, the primary treatment objectives must focus on managing the infection by addressing the underlying systemic condition and eliminating the source of infection. Antibiotic therapy should be tailored to the results of microbiological cultivation. In our study, the majority of patients were treated surgically. Conservative treatment options can effectively manage many cases; however, they may not always be sufficient. In general, surgical intervention should be considered when conservative treatment proves ineffective and when symptoms or imaging results indicate disease progression [8]. Immediate surgical intervention is essential when patients present with neurologic impairments, septic disease progression, or progressive deformity [21]. We and many other centers are opting for early surgery including cases without neurologic impairments or deformity as complications arising from immobilization and ongoing infection can be particularly severe in today’s older and comorbid population. Moreover, during the early stages of the infection, minimally invasive procedures reducing surgical trauma, intraoperative blood loss, and postoperative complications [22] may still be viable, while extensive strategies are required at later stages with significant bony destruction and deformity.

Our data revealed that patients with early mortality often presented with a more extensive infection, characterized by a higher prevalence of paravertebral manifestations, neurological deficits, and septic condition. Interestingly, only a small percentage of patients with early mortality were admitted in an unstable general condition necessitating intensive care therapy. This suggests that clinical deterioration may occur later in the disease course in a certain subset of patients, emphasizing the importance of identifying risk factors for rapid deterioration at an early stage of the disease.

For patients with early mortality, the most prevalent sources of infection were joint empyema (21.9%), urosepsis (18.8%), and leg ulcers (15.6%), while for surviving patients, the primary source of infection was a prior spinal surgery (29.4%). These data are in line with results from Lener et al. [23]: they reported that deceased patients were more frequently affected by primary acquired spondylodiscitis compared to surviving patients. In a large-scale nationwide study of 10,695 spondylodiscitis patients, Kim et al. [24] documented the most frequent concurrent infections as urinary tract infections (11.3%), intra-abdominal infections (9.4%), pneumonia (8.5%), and septic arthritis (4.6%). Moreover, they found that patients with concurrent infection had a four-fold higher mortality than those without. Kim et al. registered concurrent infections, whereas we identified the infective focus of spondylodiscitis. This explains why typical hospital-acquired infections were numerically predominant in the study by Kim et al. On the other hand, in some infections, such as endocarditis or joint empyema, retrospectively, it will never be possible to distinguish whether they are the infective focus of spondylodiscitis or concurrent infections. However, for clinical practice, this distinction is not relevant, as the treatment does not differ. In both cases, the infection must be addressed.

Known risk factors for the development of spondylodiscitis in general include advanced age, obesity, diabetes mellitus, substance abuse, immunodeficiency, and long-term steroid medication [8]. The present study identified advanced age, diabetes mellitus, previous steroid medication, higher CCI, and lower glomerular filtration rate (GFR) level at admission as significant risk factors for in-hospital death from spondylodiscitis. A recent German study reported increasing age, elevated creatinine and CRP levels, and the presence of rheumatoid arthritis as significant risk factors for in-hospital mortality from spondylodiscitis [15], whereas in the present study, CRP level at admission did not reach statistical significance. Additionally, unlike a study from Ohio that identified epidural empyema, neurologic deficits, in-hospital acquisition, and time to diagnosis as risk factors for an adverse outcome [25], our study did not find epidural empyema to be a significant factor. Contrary to findings from a Japanese study [16], in the present study, a history of malignant disease, hepatopathy, and endocarditis were not associated with an elevated risk for in-hospital death from spondylodiscitis. 

Interestingly, the bacterial spectrum did not differ between patients with early mortality and surviving patients. For both groups, the most frequently detected bacterium was *Staphylococcus aureus*, which is the most common pathogen for spondylodiscitis in Europe [26,27,28]. Other studies have reported increased mortality, complication rates, and treatment failure rates for spondylodiscitis patients with *Staphylococcus aureus* bacteremia [15,20,28,29].

Our study has several limitations. First, being retrospective, it relies on historical data, which may not be as comprehensive as those provided by prospective trials, particularly regarding comorbidities, prior medication, and prior clinical course before admission to hospital. Second, group sizes differed significantly, a fact that is inherent to the nature of the matter and cannot be overcome. Third, the follow-up period for surviving patients was not standardized. However, the primary outcome was in-hospital death compared to patients who survived this period, not long-term outcomes. Additionally, some potentially predictive factors were not considered in our analysis, either due to their perceived lack of relevance or unavailability in a retrospective setting.

However, it is important to note that one of the study’s strengths lies in its substantial sample size, encompassing consecutive patients treated at a single specialized center known for its high treatment standards, over an extended five-year period.

## 5. Conclusions

The present study demonstrates that even if admitted in a moderate clinical condition and if maximum medical treatment is applied immediately, a certain proportion of patients with spondylodiscitis die during their hospital stay. Special attention should be paid to elderly patients and to patients with diabetes or steroid medication who harbor an elevated risk for a fulminant disease with fatal consequences.

## Figures and Tables

**Table 1:** Demographic overview. Shows a comparison of the patients’ baseline characteristics. ASA = American Association of Anesthesiologists, BMI = body mass index, CCI = Charlson Comorbidity Index. A paired *t*-test (for continuous variables) and Fisher’s exact test/chi-square test (for categorical variables) were used.

	All N (%)	Surviving (S) N (%)	In-Hospital Mortality (M) N (%)	*p*-Value (S vs. M)
Total	430	384	32	
Age (y)				0.0018
Median	72.0	70.5	76.5	
Min.	30.0	30.0	62.0	
Max.	94.0	94.0	91.0	
Sex				n.s.
Male	271 (63.02)	238 (61.98)	24 (75.00)	
Female	159 (36.98)	146 (38.02)	8 (25.00)	
BMI (kg/m^2^)				n.s.
Mean	26.7	26.9	26.8	
STD	6.4	6.1	4.7	
ASA				n.a.
I	8 (1.86)	8 (2.08)	0 (0.00)	
II	117 (27.21)	111 (28.91)	4 (12.50)	
III	259 (60.23)	232 (60.42)	16 (50.00)	
IV	41 (9.53)	32 (8.33)	8 (25.00)	
V	5 (1.16)	1 (0.26)	4 (12.50)	
ASA				<0.0001
I–III	384 (89.30)	351(91.40)	20 (62.50)	
IV–V	46 (10.70)	33 (8.60)	12 (37.50)	
CCI				<0.0001
Median	4.0	4.0	6.5	
Min.	0.0	0.0	3.0	
Max.	12.0	12.0	12.0	
Alcohol abuse				n.s.
Present	24 (5.58)	22 (5.73)	2 (6.25)	
Absent	406 (94.42)	362 (94.27)	30 (93.75)	
Drugs IV				n.s.
Present	18 (4.19)	18 (4.69)	0 (0.00)	
Absent	412 (95.81)	366 (95.31)	32 (100.00)	
Diabetes mellitus				0.0004
Present	125 (29.07)	103 (26.82)	19 (59.38)	
Absent	305 (70.93)	281 (73.18)	13 (40.62)	
Hepatopathy				n.s.
Present	46 (10.70)	39 (10.16)	4 (12.50)	
Absent	384 (89.30)	345 (89.84)	28 (87.50)	
Steroids				0.0242
Present	23 (5.35)	18 (4.69)	5 (15.62)	
Absent	407 (94.65)	366 (95.31)	27 (84.38)	
Malignant disease				n.s.
Present	56 (13.02)	46 (11.98)	5 (15.62)	
Absent	374 (86.98)	338 (88.02)	27 (84.38)	

n.s. = non significant; n.a. = not applicable.

**Table 2:** Details of spondylodiscitis. Compares the details of spondylodiscitis between the different groups. Secondarily acquired was defined as prior spinal surgery on the same spinal level. CRP = C-reactive protein. A paired *t*-test (for continuous variables) and Fisher’s exact test/chi-square test (for categorical variables) were used.

	All N (%)	Surviving (S) N (%)	In-Hospital Mortality (M) N (%)	*p*-Value (S vs. M)
Total	430	384	32	
Etiology				0.0058
Endogenously	318 (73.95)	278 (72.40)	30 (93.75)	
Secondarily	112 (26.05)	106 (27.60)	2 (6.25)	
Localization				n.a.
Cervical	47 (10.93)	43 (11.20)	3 (9.38)	
Thoracic	68 (15.81)	61 (15.89)	5 (15.63)	
Lumbosacral	260 (60.47)	235 (61.20)	16 (50.00)	
Multifocal	55 (12.79)	45 (11.72)	8 (25.00)	
Unifocal	375 (87.21)	339 (88.28)	24 (75.00)	0.0477
Multifocal	55 (12.79)	45 (11.72)	8 (25.00)	
Epidural empyema				n.s.
Present	200 (46.51)	177 (46.09)	16 (50.00)	
Absent	230 (53.49)	207 (53.91)	16 (50.00)	
Paravertebral abscess				n.s.
Present	161 (37.44)	141 (36.72)	16 (50.00)	
Absent	269 (62.56)	243 (63.82)	16 (50.00)	
CRP (mg/dL)				0.0437
Median	8.1	7.5	12.2	
Min.	0.1	0.1	1.6	
Max.	50.4	50.4	41.2	
Treated surgically				0.0003
Yes	415 (96.51)	376 (97.92)	26 (81.25)	
No	15 (3.49)	8 (2.08)	6 (18.75)	

n.s. = non significant; n.a. = not applicable.

**Table 3:** Neurological examination. Shows the neurological status at admission to the hospital indicated by muscle strength grades according to the Medical Research Council (MRC) scale, with 0 representing the worst and 5 the best muscle strength grade. Fisher’s exact test was used.

	Surviving (S) N (%)	In-Hospital Mortality (M) N (%)	*p*-Value (S vs. M)
Total	384	32	
MRC			n.a.
5	250 (65.10)	15 (46.88)	
4	60 (15.63)	2 (6.25)	
3	22 (5.73)	2 (6.25)	
2	16 (4.17)	6 (18.75)	
1	12 (3.13)	1 (3.13)	
0	20 (5.21)	3 (9.38)	
n.a.	4 (1.04)	3 (9.38)	
MRC			0.0064
5–4	310 (80.73)	17 (53.12)	
3–0	70 (18.23)	12 (37.50)	
n.a.	4 (1.04)	3 (9.38)	
Bladder/bowel dysfunction			0.0054
Yes	51 (13.28)	9 (28.13)	
No	331 (86.20)	16 (50.00)	
n.a.	2 (0.52)	7 (21.88)	

n.a. = not applicable.

**Table 4:** Infective focus of the early mortality group. Depicts the infective focus of patients in the early mortality group. In six cases, more than one focus was found.

Infective Focus	N (%) 32
Joint empyema	7 (21.9)
Urogenital tract	6 (18.8)
Leg ulcers	5 (15.6)
Endocarditis	4 (12.5)
Prior spinal surgery	2 (6.3)
Pulmonal	2 (6.3)
Catheter-associated	2 (6.3)
Teeth	1 (3.1)
Ears–Nose–Throat	1 (3.1)
Not found	7 (21.9)

**Table 5:** Infective focus of the survivor group. Shows the infective focus of patients in the survivor group. In 36 cases, more than one focus was found.

Infective Focus	N (%) 384
Prior spinal surgery	113 (29.4)
Joint empyema	38 (9.9)
Urogenital tract	37 (9.6)
Teeth	36 (9.4)
Leg ulcers	29 (7.6)
Endocarditis	19 (4.9)
Prior spinal infiltration	16 (4.2)
Pulmonal	11 (2.9)
Catheter-associated	10 (2.6)
Ears–Nose–Throat	10 (2.6)
Peritonitis	3 (0.8)
Hepatic abscess	1 (0.3)
Cholangitis	1 (0.3)
Infected aortic prosthesis	1 (0.3)
Not found	95 (24.7)

## Data Availability

The data presented in this study are available on request from the corresponding author.

## Supplementary Materials

The following supporting information can be downloaded at: https://www.mdpi.com/article/10.3390/jcm12237228/s1, Table S1: Results of simple logistic regression analysis. Table S2: Results of multiple logistic regression analysis.
