# Supplementary material for: In-Hospital Mortality from Spondylodiscitis: Insights from a Single-Center Retrospective Study
Source: J Clin Med. 2023 Nov 22;12(23):7228. doi: 10.3390/jcm12237228 (PMC10707421; doi:10.3390/jcm12237228)
Supplement: Supplementary file 1 [file jcm-12-07228-s001.zip › jcm-2654669-supplementary.pdf]

Variable	B	Odds ratio	95% CI	p-value
Diabetes mellitus<br>Constant	1.383<br>-3.073	3.987<br>0.04626	1.917 - 8.542<br>0.02519 - 0.07730	0.0002
Malignant disease<br>Constant	0.3300<br>-2.527	1.391<br>0.07988	0.4539 - 3.520<br>0.05273 - 0.1158	0.5322
Steroid medication<br>Constant	1.326<br>-2.607	3.765<br>0.07377	1.173 - 10.30<br>0-04874 - 0.1068	0.0279
Alcohol abuse<br>Constant	0.09255<br>-2.490	1.097<br>0.08287	0.1707 - 3.976<br>0.05592 - 0.1181	0.9045
Endocarditis<br>Constant	1.010<br>-2.568	2.744<br>0.07671	0.7573 - 7.921<br>0.05106 - 0.1105	0.1146
Paravertebral abscess<br>Constant	0.5443<br>-2.720	1.723<br>0.06584	0.8311 - 3.575<br>0.03811 - 0.1055	0.1421
Epidural empyema<br>Constant	0.1566<br>-2.560	1.169<br>0.07729	0.5650 - 2.421<br>0.04463 - 0.1243	0.6707
Hepatopathy<br>Constant	0.2341<br>-2.511	1.264<br>0.08116	0.3605 - 3.435<br>0.05399 - 0.1170	0.6836
Age (y)<br>Constant	0.05896<br>-6.772	1.061<br>0.001145	1.024 - 1.105<br>4.845e-005 -0.01631	0.0006
BMI (kg/m^2)<br>Constant	-0.004644<br>-2.706	0.9954<br>0.06682	0.9193 - 1.066<br>0.009263 - 0.5390	0.9016
CRP (mg/dl)<br>Constant	0.03189<br>-2.917	1.032<br>0.05410	0.9993 - 1.065<br>0.02905 - 0.09423	0.0548
CCI<br>Constant	0.3681<br>-4.458	1.445<br>0.01159	1.253 - 1.681<br>0.004025 - 0.02918	<0,0001
GFR (ml/min)<br>Constant	-0.02038<br>-1.171	0.9798<br>0.3099	0.9678 - 0.9916<br>0.1370 - 0.6560	0.0008

Supplementary Table S1

Variable	B	Odds ratio	95% CI	p-value
Age (y)	-0.06477	0.9373	0.8945 - 0.9767	0.0038
Alcohol abuse	-1.224	0.2942	0.06293 - 2.117	0.1521
Diabetes mellitus	1.476	4.375	2.025 - 9.814	0.0002
Epidural empyema	0.08872	1.093	0.4938 - 2.436	0.8263
Paravertebral abscess	-0.6647	0.5144	0.2251 - 1.163	0.1099
Endocarditis	-0.8495	0.4276	0.1275 - 1.723	0.1916
Steroid medication	-1.317	0.2679	0.08633 - 0.9428	0.0281
Constant	6.884	976.6	41.68 - 38209	<0.0001

Supplementary Table S2
